# Emergence of mixed infection of Beijing/Non-Beijing strains among multi-drug resistant *Mycobacterium tuberculosis* in Pakistan

**DOI:** 10.1007/s13205-016-0423-9

**Published:** 2016-04-19

**Authors:** Samar Mustafa, Hasnain Javed, Jawairia Hashmi, Nazia Jamil, Zarfishan Tahir, Abdul Majeed Akhtar

**Affiliations:** 1 Department of Microbiology and Molecular Genetics, University of the Punjab, Lahore, Pakistan; 2 Punjab Provincial TB reference Lab, Institute of Public Health, Lahore, Pakistan

**Keywords:** *Mycobacterium tuberculosis*, Resistance, *IS6110*, Beijing, Non-Beijing, *Transposase*, CRISPR

## Abstract

Tuberculosis (TB) remains as one of the deadliest diseases after HIV globally with 95 % of deaths confined to low-and-middle income countries. Pakistan is fifth among the 22 high-burden TB countries with the incidence rate of 230/100,000 persons, however, studies related to prevalent *Mycobacterium tuberculosis* strains and their spread, drug resistance pattern and evolutionary genetics are inadequate. The present study was undertaken to highlight the circulation of *M. tuberculosis* strains causing drug resistant TB in our community by targeting the molecular marker *IS6110* and then characterization of these strains as Beijing and Non-Beijing genotypes. Sputum samples from 102 MDR TB suspects from different cities of Punjab were collected and their record was stored in a database. Sputum samples were evaluated by Ziehl Neelson staining and cultured on Lownstein Jensen medium by Modified Petroff’s method. DST was performed for first-line anti-mycobacterial drugs by indirect proportion method. *Mycobacterium tuberculosis* isolates were investigated for the presence of *IS6110* and further identification as Beijing, Non-Beijing or mixed genotype. Percentage of male and female patients was found to be 58.8 and 41.2 % respectively. DST showed resistance of 93 % of isolates to isoniazid and rifampicin. All of the isolates showed positive results for *IS6110* amplification. Based on PCR amplification of Beijing and non-Beijing primer sets 4.9 % of the patients showed infection with pure Beijing isolates, 14.7 % with both Beijing and non-Beijing isolates and 80.3 % with pure non-Beijing isolates. Analysis of *IS6110* and Beijing sequences showed the presence of putative *transposase* conserved domain while non-Beijing sequences were epitomized with RAMP_I_III superfamily domain (CRISPR-associated protein family). TB in Pakistan is predominantly caused by Non-Beijing genotypes, but Beijing strains showed incessant circulation in our community as both single and mixed (co-infecting Non-Beijing and Beijing) strains.

## Introduction

Tuberculosis (TB) continues as one of the deadliest contagious diseases across the globe. Nearly 9 million people developed TB in 2013 and the year ended with the demise of 1.5 million people due to TB. Of the total TB cases and deaths, around 56.25 % are men but the burden of the disease is eminent in women as well. To no surprise, out of the total TB cases occurred in 2014, 58 % belonged to the South-east Asian and Western Pacific regions (WHO 2015).

Pakistan stands fifth among the 22 high-burden countries and fourth among 27 MDR-TB countries. Nearly 420,000 cases occur each year out of 9000 cases are of DR-TB. Socio-economic conditions are greatly influenced by TB as 75 % of cases are among the dynamic (15–45) age group (Programme NTC 2012). TB is closely associated to poverty and social deprivation (Lönnroth et al. 2009). Poor living conditions like overcrowding, poor ventilation, close proximity and malnutrition are the major risk factors for contracting TB infection and disease. Lack of awareness to TB epidemiology, management and control in TB endemic areas worsens the situation (Narasimhan et al. 2013).

Furthermore, the rapid increase in the incidence of MDR-and XDR-TB complicated the issue. MDR, resistance to at least 2 most potent first line drugs (rifampicin and isoniazid) while extensive drug resistant tuberculosis (XDR-TB) is defined as MTB strains resistant to first line drugs and also resistant to any fluoroquinolone and at least one of the three second line injectable drugs: kanamycin (KAN), amikacin (AMK), and capreomycin (CAP) (Hamilton et al. 2007).

The timely and accurate diagnosis of drug resistant TB is utmost important for suitable intervention to halt the disease progression. It also facilitates to halt the transmission of MDR and XDR-TB strains (Lange et al. 2014). Drug susceptibility testing of MTB contains two folds importance. One patient infected with resistant bacilli can achieve a favorable response after DST results if treatment is adjusted after his DST pattern. Secondly DST results will be useful to monitor the National TB control programs efforts (Thomsen et al. 2000). Although the traditional DST has high sensitivity and specificity, it is time-consuming (6–9 weeks) which demands for rapid drug resistance detection assays.

Molecular diagnostic tools have provided the solution to this problem. Nucleic acid amplification tests (NAATs) are based on the identification and amplification of nucleic acids’ sequences that are highly specific to *Mycobacterium tuberculosis* (Drobniewski et al. 2012).

Insertion sequences are small mobile elements which are proved to be present in various bacterial genomes. *IS6110* is one of the IS elements which are intensively studied and reported to be specific to *M. tuberculosis* complex genome. *IS6110* is present in multiple copies within the *M. tuberculosis* genome which makes it an important epidemiological tool for strain identification (Menéndez et al. 2014; Siddiqui et al. 2013). The capacity to differentiate between the strains depends on the variation in copy number as well as the insertion sites of *IS6110* (Thorne et al. 2011).

Molecular epidemiological studies of tuberculosis are worthwhile in understanding the transmission and geographical pattern of drug resistant strains. Such studies have discovered multiple mycobacterial lineages worldwide (Filliol et al. 2002). Drug resistance patterns shown by genotypes of *M. tuberculosis* vary globally. Resistance pattern showed by Beijing genotype is different region-wise (Glynn et al. 2002); however, this genotype has been largely reported to be related to DR-TB outbreaks in Africa, Asia and Europe (Johnson et al. 2010; Niemann et al. 2010). Beijing genotype has been shown to be associated with MDR-TB incidence in Pakistan (Tanveer et al. 2008).

Infection and recurrence of TB have been believed to be caused by primary infection or reactivation of single strain for decades (Zheng et al. 2015). However, research in the field of molecular epidemiology has revealed the term mixed infection of multiple strains among single individuals and has got prime importance among researchers and TB Control programs (Zetola et al. 2014). Mixed Infection can enhance the ability of MTB strains to acquire additional mutations and increase the rate of treatment failure (Cohen et al. 2012).

By amplifying and detecting the DNA sequences of Beijing and Non-Beijing strains, it has been observed that many retreatment cases contain multiple strains. It has been extensively studied that the region *Rv2819* and part of *Rv2820* in *M. tuberculosis* genome is specifically deleted in all Beijing strains, while it is present in Non-Beijing strains (Khosravi et al. 2014).

As mixed infection may be particularly common in high burden drug resistant TB countries, so the aim of the present study was to elucidate the drug resistant pattern of multi-drug resistant MTB strains in our community, genotypic identification and detection of mixed infection of Beijing and non-Beijing strains in Pakistan and its prevalence in new and retreatment cases on the basis of deleted and intact genetic regions in Beijing and Non-Beijing strains of *M. tuberculosis* respectively.

## Materials and methods

### Patients and specimens

Sputum samples of 102 patients suspected of MDR-TB were collected from different PMDT (Programmatic Management of Drug resistant TB) sites during a period of 5 months (August 2014 to December 2014). After taking informed consents, a single Sputum sample from each patient was collected according to the guidelines provided by WHO.

### Sample processing and culturing

For preliminary detection of MTB Isolates, all sputum samples were inoculated on Lowenstein Jensen medium after being digested and decontaminated by Modified Petroff’s method using 4 % NaOH solution and then concentrated by centrifugation at 3000*g* for 30 min (Kent and Kubica 1985; Tripathi et al. 2014). Concentrated samples were then used for smearing and culturing (Cappuccino and Sherman 2014).

### Smear microscopy

Each sample was used for smearing and then checked for the presence of acid fast bacillus by Ziehl Neelson staining (Cappuccino and Sherman 2014).

### Drug susceptibility testing

Standard drug susceptibility testing (DST) of first line drugs using 1 % indirect proportion method on solid Lowenstein–Jensen medium (Canetti et al. 1969) was performed on positive cultures only. H37Rv strain was used as control. DST was conducted using LJ medium infused with isoniazid (Inh), rifampin (Rif), ethambutol (Emb), and streptomycin (Stm). The antibiotic concentrations in the medium were 20 μg/ml for Inh, 40 μg/ml for Rif, 2 μg/ml for Emb, and 10 μg/ml for Stm.

### Genomic DNA isolation and gel electrophoresis

Genomic DNA of drug resistant MTB strains was extracted by the CTAB method (Amaro et al. 2008). A loopful of mycobacterial culture from LJ slope was taken and transferred to eppendorfs containing low T.E buffer. Inactivate the mycobacterial cells by heating at 80 °C for 1 h. After cooling to room temperature, bacterial suspension cultures were incubated overnight at 37 °C with lysozyme then incubated them with SDS and Proteinase K at 65 °C for 20 min following incubation with pre-warmed CTAB/NaCl solution at same temperature for 10 min. Finally, the DNA was precipitated by using phenol–chloroform solution and purified by using ethanol. DNA isolation was confirmed by visualizing the bands on 0.9 % agarose gel.

### PCR for the detection of *IS6110*

The presence of *IS6110* specific for *M. tuberculosis* in all isolates was tested by using primer set ISF 5′CCTGCGAGCGTAGGCGTCGG3′ and ISR 5′CTCGTCCAGCGCCGCTTCGG 3′. PCR amplicon obtained by using same primers from DNA isolated from H37Rv standard strain was used as positive control whereas for the negative control PCR reaction mixture without any DNA was used.

### PCR for the detection of Beijing and Non-Beijing strains

As previously mentioned that region spanning genes *Rv2816* to *Rv2819* and part of *Rv2820* is missing in all Beijing strains so for detection of Beijing strains primer set BF 5′ACCGAGCTGATCAAACCCG 3′ and BR 5′ ATGGCACGGCCGACCTGAATGAACC 3′ for amplification of 239-bp fragment containing region specific part of *Rv2819* and part of *Rv 2820* was used. While for the detection of Non-Beijing strains, amplification of 539-bp PCR fragment was amplified using primer set 5′GATCGCTTGTTCTCAGTGCAG3′and NonBR 5′CGAAGGAGTACCACGTGGAG 3′ containing region specific for *Rv2819*.

### PCR product purification, sequencing and bioinformatics

PCR products were purified by using GeneJET™ Purification kit (#K0701). PCR products were sequenced by Sanger sequencing method from AB sequencer (Applied Bioscience). Sequencing results were analyzed using the following software tools: BLAST (http://www.ncbi.nlm.nih.gov/BLAST/) MEGA6 (http://www.megasoftware.net/) SEQUIN (http://www.ncbi.nlm.nih.gov/Sequin/).

## Results

### Demographic information

A total of 102 suspected MDR-TB samples were collected from 6 big cities of Punjab Province. Samples were obtained from both males and female patients with mean age of 33.3 years. Proportion of male and female patients was 58.8 % (mean age 33.3) and 41.2 % (mean age 33.2), respectively. Incidence of Drug resistant TB in our study group was found to be higher in age group of 30–35 years. Samples included in the study group were divided into two major subsets, that is, new and retreatment cases (Persons who default from the initial treatment and fail to improve, or relapse after the initial treatment Becerra et al. 2000) on the basis of their treatment status.

### Drug-susceptibility pattern

Out of the total 102 isolates, 95 (93 %) showed resistance to isoniazid and rifampicin and considered as MDR. Among 95 MDR samples, 44 were new cases and 51 were retreatment cases Resistance to streptomycin was found to be greater than ethambutol. 51 % of the isolates showed resistance to all four first-line drugs (Table 1).

**Table 1:** Percentage of resistance to FLDs among new and retreatment cases

First-line drug(s)	No. (%) of new cases resistant to the drug(s) (*n* = 51)	No. (%) of retreatment cases resistant to the drug(s) (*n* = 51)	Total no. (%) of cases (*n* = 102)
Inh	47 (92 %)	51 (100 %)	98 (96 %)
Rif	47 (92 %)	51 (100 %)	98 (96 %)
Inh, Rif	44 (86.2 %)	51 (100 %)	95 (93 %)
Inh, Rif, Emb	22 (43.14 %)	38 (74.5 %)	60 (58.8 %)
Inh, Rif, Stm	26 (50.98 %)	41 (80.4 %)	67 (65.6 %)
Inh, Rif, Emb, Stm	20 (39.22 %)	32 (62.74 %)	52 (50.9 %)

*FLD* first-line drugs

### PCR amplification of *IS6110*

PCR amplification using primers targeting *IS6110* showed positive amplification with 100 % of the total (102) isolates (Fig. 2).

### PCR based detection of Beijing and Non-Beijing strains

**Fig. 1:**
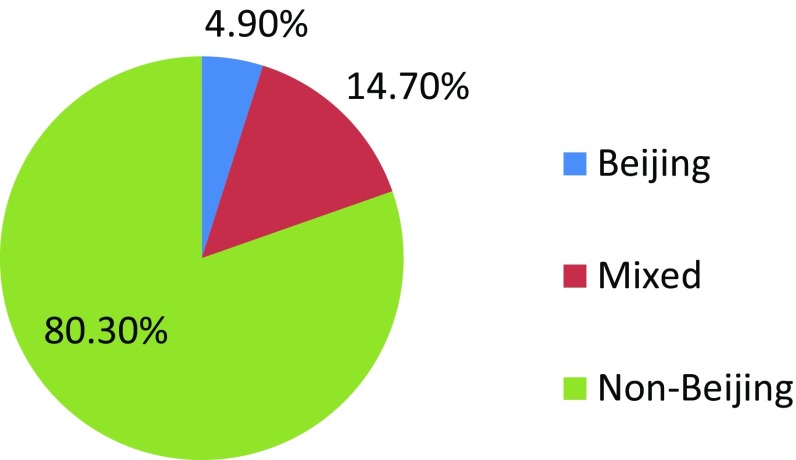
Magnitude of different genotypes of *M. tuberculosis* among the study isolates

**Fig. 2:**
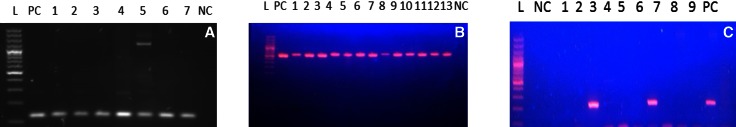
Division of polymerase chain reaction (PCR) amplified products. **a** PCR amplification with primer set 1 (ISF and ISR; Product size ~124 bp). **b** PCR amplification with primer set 2 (NB-F and NB-R; Product size 569 bp). **c** PCR amplification with primer set 3 (BF and BR; Product size 239 bp). L: 100 bp molecular marker; Arabic numerals: clinical samples; NC: negative control (in this case ddH_2_O)

Among 102 collected isolates 4.9 % of the isolates showed positive amplification with the primer set targeting Beijing-specific sequence while for non-Beijing amplification showed positive for 80.3 % of the isolates. Mixed infection with both Beijing and Non-Beijing strain has also been detected in 14.7 % of the patients (Fig. 1) as the DNA isolated from their cultures showed positive amplification with Beijing and non-Beijing primer sets (Fig. 2).

Analysis of new and retreatment cases showed that non-Beijing strains contribute the largest proportion of our isolates (Table 2).

**Table 2:** Frequency of different types of infections among new and retreatment MDR cases

Type of infection	No. (%) of new cases (*n* = 44)	No. (%) of retreatment cases (*n* = 51)	Total no. (%) of cases (*n* = 95)
Beijing	3 (6.8 %)	2 (3.9 %)	5 (5.3 %)
Non-Beijing	29 (65.9 %)	46 (90.2 %)	75 (78.9 %)
Mixed	12 (27.2 %)	3 (5.9 %)	15 (15.7 %)

### Sequence analysis

**Fig. 3:**
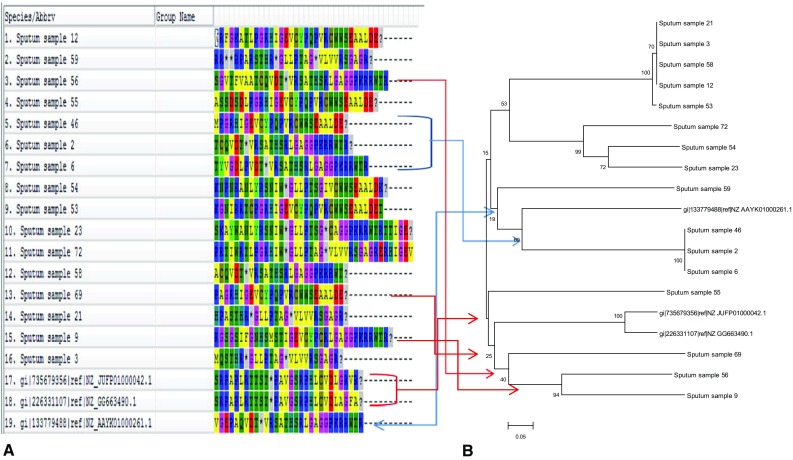
Comparison of genetic clusters on the basis of translated protein sequences.** a** Aligned IS6110 sequences: cluster 1 (46, 2, 6) is indicated with* blue arrows*; cluster 2 (69, 56, 9) is indicated with* red arrows*.** b** *Phylogram of the* M. tuberculosis* sequences on the basis of IS6110. *The evolutionary history was inferred using the Neighbor-Joining method. The optimal tree with the sum of branch length = 3.79170867 is shown. The percentage of replicate trees in which the associated taxa clustered together in the bootstrap test (100 replicates) are shown next to the branches. The tree is drawn to scale, with branch lengths in the same units as those of the evolutionary distances used to infer the phylogenetic tree. The evolutionary distances were computed using the p-distance method and are in the units of the number of base differences per site. The analysis involved 19 nucleotide sequences. All positions containing gaps and missing data were eliminated. There were a total of 62 positions in the final dataset. Evolutionary analyses were conducted in MEGA6

**Fig. 4:**
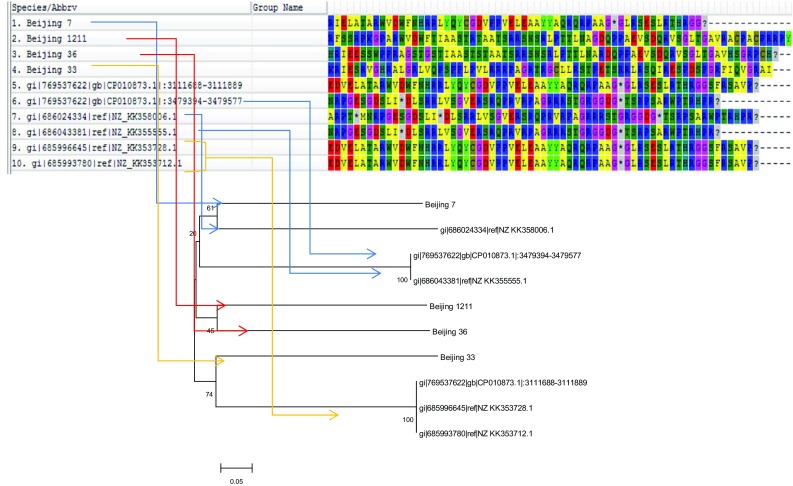
Comparison of genetic clusters on the basis of translated protein sequences.** a** Aligned Beijing sequences: cluster 1 (Beijing 7) is indicated with* blue arrows*; cluster 2 (Beijing 1211, 36) is indicated with* red arrows*; cluster 3 (Beijing 33) is indicated with* orange arrows*.** b** *Phylogram of the* M. tuberculosis* sequences on the basis of Beijing-specific sequences. *The evolutionary history was inferred using the Neighbor-Joining method. The optimal tree with the sum of branch length = 2.46910112 is shown. The percentage of replicate trees in which the associated taxa clustered together in the bootstrap test (100 replicates) are shown next to the branches. The tree is drawn to scale, with branch lengths in the same units as those of the evolutionary distances used to infer the phylogenetic tree. The evolutionary distances were computed using the* p*-distance method and are in the units of the number of base differences per site. The analysis involved 10 nucleotide sequences. Codon positions included were 1st + 2nd + 3rd + Noncoding. All positions containing gaps and missing data were eliminated. There were a total of 178 positions in the final dataset. Evolutionary analyses were conducted in MEGA6

**Fig. 5:**
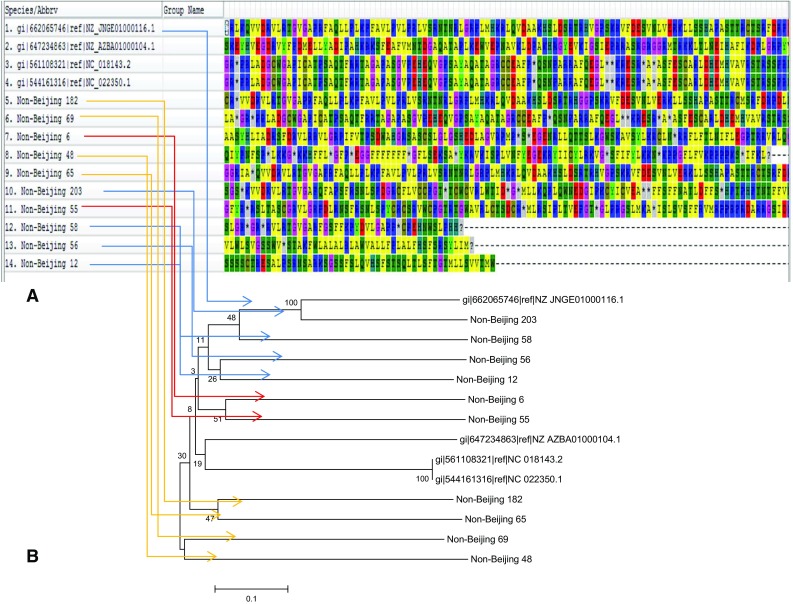
Comparison of genetic clusters on the basis of translated protein sequences. ** a** Aligned Non-Beijing sequences cluster 1 (Non-Beijing 203, 58, 56, 12) is indicated with* blue arrows*; cluster 2 (Non-Beijing 6, 55) is indicated with* red arrows*; cluster 3 (Non-Beijing 182, 65, 69, 48) is indicated with* orange arrows*.** b** *Phylogram of the* M. tuberculosis* sequences on the basis of non-Beijing specific sequences. *The evolutionary history was inferred using the Neighbor-Joining method. The optimal tree with the sum of branch length = 4.40932938 is shown. The percentage of replicate trees in which the associated taxa clustered together in the bootstrap test (100 replicates) are shown next to the branches. The tree is drawn to scale, with branch lengths in the same units as those of the evolutionary distances used to infer the phylogenetic tree. The evolutionary distances were computed using the* p*-distance method and are in the units of the number of base differences per site. The analysis involved 14 nucleotide sequences. Codon positions included were 1st + 2nd + 3rd + Noncoding. All positions containing gaps and missing data were eliminated. There were a total of 137 positions in the final dataset. Evolutionary analyses were conducted in MEGA6

BLAST results based on *IS6110* showed 100 % sequence similarity with putative *transposases* of *M. tuberculosis* isolates. Analysis of Beijing sequences with NCBI BLAST showed 98 % sequence similarity with *transposase* of *M. tuberculosis* Beijing-like strain (GenBank: CP010873.1) while non-Beijing sequences showed 99 % sequence similarity with CRISPR-associated protein Csm5 of *M. tuberculosis*. 3 of the non-Beijing sequences have been submitted to the Genbank and their accession numbers are KR362602 (Non-Beijing 55), KR362601 (Non-Beijing 65) and KR362600 (Non-Beijing 69). Phylogenetic analysis revealed similarity with reference strains as well as among the strains themselves (Figs. 3, 4, 5).

## Discussion

Acid-fast bacilli (AFB) microscopy and mycobacterial culturing have been practiced for years for the lab diagnosis of *M. tuberculosis*. But, being labour intensive and time consuming, these methods demand for rapid, more efficient and reliable methods for TB diagnosis. Timely and accurate diagnosis is crucial to the commencement of effective antimycobacterial therapy of TB cases. PCR is greatly used for the detection of *M. tuberculosis* either using sputum or the cultures obtained from sputum (Huang et al. 2010; Kaul 2001; Soini and Musser 2001).In case of smear positive pulmonary TB, diagnosis is confirmed or dismissed in just 48 h using PCR rather than 2–8 weeks for culturing (Balamurugan et al. 2006). Newer molecular methodologies have identified extensive genetic diversity among *M. tuberculosis* isolates thereby making it quite difficult to classify different disease causing strains precisely (Van Embden et al. 1993). In the present study, PCR amplification targeting genetic markers specific to distinct evolutionary lineages was utilized to differentiate *M. tuberculosis* isolates on the basis of presence or absence of these markers. This method provided us with a simplified approach to estimate the prevalence of Beijing and Non-Beijing strains of *M. tuberculosis* in our community.

Our results showed that overall drug resistance is higher in retreatment cases. Complete resistance to both isoniazid and rifampicin in 93 % of the samples confirmed the isolates as MDR. In addition, resistance to other FLDs was found to be significant among both new and retreatment cases.

*IS6110* is considered as ideal target in epidemiological studies of *M. tuberculosis* because it is randomly distributed and present in multiple copies in the MTB genome. Many studies claimed the usefulness of PCR based techniques in identification of MTB using IS6110 as a target (Doroudchi et al. 2000; Siddiqui et al. 2013), and our study confirmed these results by showing high degree of sensitivity of PCR based detection of MTB.

In our study results, most strains belong to the non-Beijing family i.e. 70.6 % of the new and 90.2 % of the retreatment cases. The higher percentage of non-Beijing strains was found in accordance with another study carried out in Pakistan suggesting the higher prevalence of Central Asian strain (CAS1) family of *M. tuberculosis* rather than Beijing genotype (Hasan et al. 2006). Another study from Punjab (Pakistan) showed that non-Beijing isolates (91.1 % of the total isolates) are more prevalent than Beijing isolates (8.9 %) in the region (Arif and Hussain 2014). Furthermore, mixed infection with Beijing and non-Beijing strains constitutes the second highest proportion in our study. However, the percentage of mixed infection was found to be higher in new cases as compared to retreatment cases. The existence of mixed infection in ~23 % of the new cases may suggest that reinfection is common in a high burden country like Pakistan. A speculation can be made that a continuing TB infection in an individual with one strain may weaken the immune response thus increasing the risk of super infection with a different strain before advancing to a single disease episode. Another proposition is that the super infection after some time of the primary infection may trigger the disease development and activation of the initial infection. Previous reports proposed that a significant portion of first episodes of disease is the probable result of reinfection which depends on indigenous disease prevalence and extent of protection given by an earlier infection (Cohen et al. 2007, 2012; Du Plessis et al. 2001; Wang et al. 2015). Although the percentage of Beijing strains was less significant in comparison with non-Beijing strains, but their higher frequency among new cases as both single infecting strain or causing mixed infection with other strains may suggest that they are continually circulating and evolving in our environment. Molecular diagnostic tools are vital to identify and characterize drug-resistant *M. tuberculosis* strains. In a high-burden and low-resource setting, like Pakistan PCR-based approaches provide rapid diagnosis of drug-resistant TB and insights on the prevalent MTB genotypes in the region. Non-Beijing strains are mostly prevalent in this region but continual circulation of Beijing strains has also been indicated. Furthermore, in silico methods aid in precise characterization of these strains by nucleotide and amino acid sequence analysis.
